# Regulation of yak *longissimus lumborum* energy metabolism and tenderness by the AMPK/SIRT1 signaling pathways during postmortem storage

**DOI:** 10.1371/journal.pone.0277410

**Published:** 2022-11-28

**Authors:** Yayuan Yang, Jieyuan Yang, Qunli Yu, Yongfang Gao, Ya Zheng, Ling Han, Xuezhi Ding

**Affiliations:** 1 Key Laboratory of Veterinary Pharmaceutical Development, Ministry of Agricultural and Rural Affairs, Lanzhou Institute of Husbandry and Pharmaceutical Sciences of Chinese Academy of Agricultural Sciences, Lanzhou, China; 2 School of New Energy and Power Engineering, Lanzhou Jiaotong University, Lanzhou, Gansu, PR China; 3 College of Food Science and Engineering, Gansu Agricultural University, Lanzhou, Gansu, PR China; 4 Gansu Innovation Center of Fruit and Vegetable Storage and Processing, Agricultural Product Storage and Processing Research Institute, Gansu Academy of Agricultural Sciences, Lanzhou, China; 5 Laboratory of Agricultural & Food Biomechanics, Institute of Biophysics, College of Science, Northwest A&F University, Yangling, Shaanxi, China; University of Guelph Ontario Agricultural College, CANADA

## Abstract

AMPK can activate nicotinamide phosphoribosyltransferase (NAMPT), increasing the ratio of oxidized nicotinamide adenine dinucleotide (NAD^+^)/reduced nicotinamide adenine dinucleotide (NADH) ratio, leading to the activation of the energy receptor SIRT1. This pathway is known as the AMPK/SIRT1 signaling pathway. SIRT1 deacetylates and activate LKB1, which is activated by phosphorylation of AMPK (Thr172) and inhibited by phosphorylase-mediated dephosphorylation of AMPK. At the same time, increased AMP/ATP and NAD^+^/NADH ratios lead to the activation of AMPK and SIRT1. SIRT1 and AMPK can activate each other forming a positive feedback loop, which can strengthen catabolism and weaken anabolism thus maintaining energy homeostasis of energy metabolism. At present, there has been no systematic study on AMPK-associated signaling cascades in stored yak meat and details of the AMPK/SIRT1 signaling under these conditions are not known. In this study, NAD^+^, NADH were added to yak *longissimus thoracic* muscles to study AMPK pathway regulation by AMPK/SIRT1 signaling. NAD^+^ significantly increased the activity of AMPK and glycolysis during postmortem maturation, increased the rate of energy metabolism, and increased the expression of AMPK protein, indicating that NAD^+^ increased energy metabolism in the stored muscle by promoting AMPK activity. NADH treatment inhibited both AMPK activation and glycolysis, together with increasing the pH in the muscle. The results showed that SIRT1 activation elevated the activity of AMPK, leading to its phosphorylation and the activation of glycolysis. Thus, AMPK activity was found to increase in yak meat as an adaptation to hypoxic conditions. This allows more effective regulation of energy production and improves the tenderness of the meat.

## Introduction

AMP-activated protein kinase (AMPK) is closely involved in energy metabolism as it can sense and respond to changes in energy metabolism leading to the regulation of gene expression and modulating the activities of downstream molecules [1]. AMPK is a heterotrimer composed of one catalytic subunit (α) and two regulatory subunits (β, γ). It has serine/threonine kinase activity. The activity of AMPK is regulated by the competitive binding of AMP and ATP to the γ subunit. Heat restriction or ATP consumption during exercise leads to an increase in the AMP/ATP ratio, activating AMPK [2]. AMPK can activate NAMPT (nicotinamide phosphoribosyltransferase) and increase the NAD^+^/NADH ratio, leading to the activation of another energy receptor SIRT1. This pathway has, therefore, been named the AMPK/SIRT1 signaling pathway [3]. In addition, SIRT1 can deacetylate and activate liver kinase B1 (LKB1) which activates AMPK by phosphorylating AMPK (at Thr172) and inhibiting the AMPK dephosphorylation by phosphorylase [4]. This indicates that increases in the AMP/ATP and NAD^+^/NADH ratios lead to AMPK and SIRT1 activation during exercise or heat restriction [5]. In addition, both AMPK and SIRT1 can activate each other to form a positive feedback loop, thus strengthening catabolism and weakening anabolism, which is helpful to maintain energy metabolism homeostasis. SIRT1 is not only indirectly regulated by AMPK but also regulated by other kinases [6]. During oxidative stress, JNK (c-Jun N-terminal kinase) interacts with SIRT1 to phosphorylate Ser27, Ser47, and Thr530 on SIRT1, thus enhancing enzyme activity and nuclear localization [7]. In cancer cells, mTOR phosphorylates Ser47 and inhibits SIRT1, although whether this occurs in skeletal muscle cells is not known [8]. It is possible that the AMPK/SIRT1 signaling pathway may be involved in the quality control of skeletal muscle by regulating apoptosis, protein synthesis, and degradation [9].

AMPK and SIRT1 coordinate the expression of genes related to skeletal muscle energy metabolism. Activated AMPK indirectly activates SIRT1 by increasing the NAD^+^/NADH ratio in the cell, thereby promoting the expression of its downstream target genes (such as PGC-1a, Ctp1, Pdk4, and Glut4) [10]. At the same time, SIRT1 can deacetylate, and thus activate, LKB1 [10]. Activated LKB1 then phosphorylates the Thr172 site on AMPK to activate AMPK. Lin et al. showed that the PGC-1α gene is closely related to the transformation of muscle fibers and that over expression of the gene can increase the content of type I muscle fibers in skeletal muscle [11]. AMPK is activated by external stimulation to increase the NAD^+^/NADH ratio in cells, leading to the activation of SIRT1 [12]. Activated SIRT1 can mediate the deacetylation and activation of the downstream target PGC-1α, while P-AMPK can also directly phosphorylate Thr177 and Ser538 on PGC-1α, thereby activating PGC-1α [12]. Activated PGC-1α promotes the formation of type I muscle fibers by regulating the activities of its downstream target genes MEF2 and nuclear respiratory factor 1 (Nrf1) [13]. MEF2 can also bind directly to the promoter of the PGC-1α gene to regulate the expression of PGC-1α, thereby promoting the conversion of muscle fiber types [13]. Cantó C and other studies have shown that deacetylation of SIRT1 and PGC-1a is necessary for the AMPK-mediated enhancement of PGC-1α activity, and inhibition of SIRT1 activity will reduce deacetylation of PGC-1α induced by 5-aminoimidazole-4-carboxamide ribonucleotide (AICAR) [14]. AMPK regulation of genes related to mitochondrial and lipid metabolism is largely dependent on the regulation of PGC-1α activity by SIRT1 [14].

Although there have been many studies on AMPK and SIRT1 in recent years, most have focused on cell senescence and survival. The roles of these factors in the quality control of skeletal muscle remain to be determined. Studies have shown that the AMPK/SIRT1 signaling pathway is involved in the regulation of protein synthesis, proliferation and decomposition, autophagy, cell degradation, and apoptosis, together with other cellular processes, and may thus also be associated with the maintenance of quality in skeletal muscle. What is the expression level of AMPK/SIRTl signaling pathway-related proteins in skeletal muscle cells during postmortem maturation? What is the effect of protein expression on the energy metabolism of skeletal muscle after slaughtering? What is the role and mechanism of high-altitude yak meat under hypoxic adaptation? These issues require further research.

## Materials and methods

### Materials

All procedures involved in animal care and their use were in strict accordance with the guidelines for the Care and Use of Laboratory Animals, Lanzhou Institute of Husbandry and Pharmaceutical Sciences, CAAS, China [SYXK-2019-0012].

Ten healthy yaks (average age: 3 yr; average body weight: 240–280 kg) maintained under the same natural grazing conditions (2500 m altitude) were obtained from the Tibetan Autonomous Prefecture of Gannan, Gansu Province, China. They were slaughtered humanely at a commercial meat processing company (Gansu Dahe Ecological Food Co. Ltd, Gansu, China). This study was conducted according to the “Operating Procedures of Cattle Slaughter” of the National Standards of the People’s Republic of China, including specific conditions for animal welfare (SAC/TC516). After slaughter, the *longissimus thoracic* (LT) muscles (from the 12th thoracic vertebra to the 5th lumbar vertebra) were immediately removed from the carcasses. After removal of all visible fat and connective tissue, 15-g samples were taken from the excised LT and immediately frozen in liquid nitrogen; this was designated as the 0-h sample. Additional samples (90 g each) were vacuum packed into pouches and transferred to the laboratory in ice bags within 90 min. The remainder of the muscle was subdivided into two fractions, one of which was used as the untreated/control group while the other (treatment group) was injected with NAD^+^ (100 μmol/L) (Sigma) and NADH (100 μmol/L) (Sigma) in the ratio 1:1 (w/v) (meat/buffer), and subsequently aged at 4°C for 12, 24, 72, 120, and 168 h. The samples analyzed at each time point were handled individually and were only used for NAD^+^ and NADH treatment. The analysis included the assessment of pH levels [13], lactic acid concentrations [15], ATP, ADP, AMP, and IMP activities [15], AMPK activity [15], morphology using confocal laser scanning microscopy (CLSM) [16], the Warner-Bratzler shear force [17], the reactive oxygen species (ROS) contents [21], and the myofibril fragmentation index (MFI) [17].

### pH measurement

A portable pH-meter with a SenvenGo (Mettler-Toledo, Switzerland) combination electrode (PN-ISO 2917, 2001) was used to measure the pH of the muscle tissue pH at 0, 12, 24, 72, 120, and 168 h [13].

### Lactic acid concentration

Five hundred milligrams of frozen muscle samples were homogenized in 500 ml 0.9% saline and centrifuged at 4200°C for 10 min. After 1:50 dilution of the supernatant with saline, the lactate content was measured using a standard commercial kit from Blue Gene Biotechnology Co. (Shanghai, China, ID:E11L0004, Specifications: 96 tests) and the absorbances at 450 nm were measured using a microplate reader. The concentrations were calculated from a calibration curve of known standard concentrations [15].

### ATP, ADP, AMP, and IMP activity

As described in Hou’s method, approximately 3 g of frozen muscle in 10 mL of PBS, pH 7.4, was centrifuged for 10 min at 15 000×g [15]. The supernatant was mixed with 1.44 ml 0.85 m K_2_CO_3_ and filtered through a 0.2-μm membrane. The concentrations of ATP, ADP, AMP, and IMP were measured by Agilent 1100 chromatography (Agilent Technologies, Santa Clara, CA, USA) at a wavelength of. A reverse-phase C18 column with a flow rate of 1 ml/min was used for quantitative analysis based on the retention time and peak area [15].

### AMPK activity

The muscle homogenate was centrifuged at 13 000 g at 4°C for 5 min. Ten microliters of the supernatant were incubated at 37°C and pH 7.0 for 10 min with the following: 0.2 mM ATP+2 μCI[32P]ATP, 0.2 mM AMP, 5 mM MgCl_2_, 0.2 mM SAMS peptide, 80 mM NaCl, 0.8 mM dithiothreitol, 0.8 mM EDTA, 8% (w/v) glycerin, and 40 mM 4-2-hydroxyethyl-1-piperazine ethane sulfonic acid in a final volume of 50 μl. Twenty microliters of the mixture were placed on Whatman filter paper (Maidstone, UK), and cut into 2 cm×2 cm sections. The filter paper sections were washed six times with 1% phosphoric acid to remove ATP and were immersed in 3 ml of scintillator (Fisher Scientific, Waltham, MA, USA) [15].

### Confocal laser scanning microscopy (CLSM)

After staining with 2-[4-(dimethylaminostyryl]-t-picoline iodide (DASPMI) as described by Andoyo et al, the morphology of the mitochondria was examined by CLSM. The meat sample (500 mg) was placed in 2 ml of dye solution at 20°C on a rotating shaker for 20 min. After removal of the staining solution, the meat samples were washed three times with 1 ml of phosphate buffer for 5 min each time, air-dried for 5 min, and placed on a glass slide with a coverslip [16].

### Warner-Bratzler shear force

Warner-Bratzler shear force (WBSF) measurements of the cooked meat (2.54 cm thick) samples were conducted as previously described by Koohmaraie, Shackelford, and Wheeler (1996). Briefly, transverse LL muscle (100 g) sections were cooked in a water bath until the center was heated to 70°C, after which they were cooled to below 30°C. Core samples (1.27 cm, parallel to longitudinal fibers) were then extracted from each LL sample, and the peak force was measured with a V-shaped shear blade with a cross-head speed of 400 mm/min [17].

### Immunoblotting

Samples were homogenized on ice with a Polytron homogenizer (IKA Works, Inc., Wilmington, NC, USA) at the highest speed for 10 s. The tissue was homogenized with 500 ml of pre-chilled (4˚C) buffer containing 20 mM Tris-HCl, 2% sodium lauryl sulfate, 5 mM EGTA, 5 mM EDTA, 1 mM DTT, 100 mM NaF, 2 mM sodium vanadate, 10 mg/ml pepsin inhibitor, 0.5 mM phenylmethylsulfonyl fluoride (PMSF), pH 7.4 [18–20]. Fifty-microgram samples were homogenized with 0.5 M Tris-HCl (pH 6.8), 2% (v/v) 2-mercaptoethanol, 20% glycerol, 4.4% (w/v) SDS, and 0.01% bromophenol blue and were boiled for 5 min with an equal volume of 2× SDS-PAGE loading buffer. Samples were added to a 5–20% gradient gel and electrophoresed in a BioRad microgel system (Hercules, CA, USA) and the proteins were transferred to nitrocellulose membranes in a buffer containing 20 mM Tris, 0.1% sodium lauryl sulfate, 20% methanol, and 192 mM glycine. The membrane was blocked in 5% fat-free milk powder in TBS/T (150 mM NaCl, 50 mM Tris-HCl [pH 7.6], and 0.1% Tween-20) for 1 h and incubated with either the primary antibody, anti-phospho-AMPKα (Cell Signaling Technology, Danvers, MA, USA) (1:500) or monoclonal PGC-1α antibody (Sigma–Aldrich, St Louis, MO, USA) (1:500) and incubated overnight. The membranes were washed with TTBS thrice and incubated with the secondary antibody: anti-rabbit IgGs conjugated with Alexa Fluor 568 (111-165-144, West Grove, PA, USA) (1:3000) for an hour at room temperature. The membranes were washed with TTBS three times again. Image scanner II and image quantification TL software were used to quantify the band densities [18]. The treated samples were then analyzed on a separate gel to reduce differences between blots. The reference band density was used to normalize the band density of different proteins. In addition, the densities of the AMPK and PGC-1α bands were used to normalize the band densities [20].

### Measurement of mitochondrial ROS

This was measured as previously described [21], with some modifications using a fluorescent indicator (DCFH-DA, Sigma). The purified mitochondria (0.1 mg/mL protein) were added to ice-cold potassium phosphate buffer (50 mm, pH 7.4) containing 5 mm DCFH-DA. The total volume of the suspended mitochondrial particles was maintained at 3.0 mL. DCFH-DA was incorporated into the membrane by pre-incubating at 37°C for 15 min. To remove the unloaded DCFH-DA, the mixture was centrifuged at 12 500 g and 4°C for 8 min. Subsequently, the fluorescence intensity (Ex = 488 nm, Em = 525 nm) was measured with a fluorescence spectrophotometer (Shimadzu RF5301, Kyoto, Japan).

### Myofibril fragmentation index (MFI)

The measurement of myofibril fragmentation index (MFI) was performed as described by Delgado et al, with a slight modification [17]. Samples were treated with 8 mL buffer (20 mmol/L K_3_PO_4_, 100 mmol/L KCl, pH 7.1; 1 mmol/L MgCl_2_, 1 mmol/L EDTA and 1 mmol/L NaN_3_) at 1 000×g at 4°C for 15 min, and the supernatant was decanted. The pellet was resuspended in the same buffer, recentrifuged, and filtered in 10 ml buffer using a sieve. The protein concentration was determined by the biuret method using BSA standards. The final protein concentration was determined to be 0.5 mg/mL, and the absorbance was measured with an ultraviolet spectrophotometer (UV2550, Shimadzu Corporation, Kyoto, Japan) at 540 nm. The reading was multiplied by 200 to calculate the magnetic field strength.

### Data analysis

One-way analysis of variance (ANOVA) was performed using SPSS software (Version 19.0, IBM Corp., Armonk, NY, USA) to analyze differences. Duncan’s multiple range test was used for significance testing between groups. A p-value of<0.05 was considered statistically significant. Origin 8.0 software was used to draw dynamics and graphics. Each experiment was repeated at least three times.

## Results and discussion

### Effects of NAD^+^ and NADH treatment on the intramuscular environment of yak meat during ripening

The pH value is used as a direct reflection of the content of acid substances (mainly lactic acid) produced by the body. It can be seen from Table 1 that, with the extension of ripening time, the pH values of both the control and treatment groups first decreased and then increased (P < 0.05). After 72 h of maturation, the pH values in the three groups decreased to the lowest values (NAD^+^ group: 5.59, Control group: 5.46, and NADH group: 5.65). Compared with the control group, the pH value of the NAD^+^ group decreased by 0.12 (P < 0.05), while the pH value of the NADH group increased by 0.10. At 72–168 h, the pH in the NAD^+^ group was significantly lower than that in the control group (P < 0.05), although there was no significant difference between the NADH group and the control group. At the same time, the rate of pH decrease was faster than that of the control group, indicating that the presence of NAD^+^ shortened the ripening time of the postmortem beef and accelerated the glycolysis process. This may be because glycogen decomposition resulting in the production of a large amount of lactic acid accelerated the acidity, evident in a pH close to the isoelectric point of myosin. Increasing H^+^ concentrations, however, may inactivate glycolytic enzymes, causing a decrease in the rate of glycolysis and an increase in pH values.

**Table 1:** Changes of glycolysis index in the process of maturation of slaughtered yak.

Time	0h	12h	24h	72h	120h	168h
Muscle glycogen ng/ml						
**NADH**	5.45±0.12^A^	4.89±0.03^B^	4.33±0.06^Cy^	3.36±0.05^Dy^	2.53±0.80^Ez^	2.01±0.03^Fy^
**Control**	5.62±0.04^A^	4.83±0.22^B^	4.36±0.02^Cy^	3.35±0.06^Dy^	2.47±0.06^Ey^	1.95±0.07^Fy^
**NAD** ^**+**^	5.55±0.24^A^	4.69±0.05^B^	4.31±0.05^Cx^	3.11±0.05^Dx^	2.41±0.05^Ex^	1.86±0.07^Fx^
**Free glucose mmol/L**						
**NADH**	9.64±0.203	9.32±0.024	9.35±0.046	9.26±0.026	9.68±0.054	9.32±0.024
**Control**	9.53±0.048	9.56±0.030	9.86±0.040	9.85±0.059	9.84±0.068	9.61±0.081
**NAD** ^**+**^	9.75±0.102	8.74±0.080	8.83±0.058	8.61±0.45	8.77±0.078	8.75±0.075
**pH**						
**NADH**	6.56±0.02^A^	5.69±0.02^B^	5.63±0.02^BCy^	5.69±0.11^Cz^	5.72±0.05^BCz^	5.76±0.04^Bz^
**Control**	6.53±0.02^A^	5.72±0.06^B^	5.62±0.06^BCy^	5.55±0.23^Cy^	5.61±0.06^BCy^	5.68±0.05^By^
**NAD** ^**+**^	6.56±0.02^A^	5.70±0.06^B^	5.59±0.07^Cx^	5.43±0.17^Dx^	5.56±0.05^CDx^	5.54±0.04^Cx^
**Lactic acid ng/ml**						
**NADH**	97.53±1.34^E^	128.36±3.28^Dx^	153.72±4.21^Bx^	204.31±6.32^Ax^	144.23±5.34^Bx^	123.58±4.52^Cx^
**Control**	97.71±1.65^E^	130.71±4.27^Dxy^	165.97±3.36^Bxy^	226.53±6.00^Ay^	157.08±4.23^By^	138.17±6.17^Cy^
**NAD** ^**+**^	97.61±1.65^E^	138.82±4.36^Dy^	176.89±5.42^By^	231.47±5.95^Az^	168.03±6.35^Bz^	156.28±3.12^Cz^

NOTE: One-way ANOVA was used for statistical analyses between the control group and two treatment groups at 0 h to 168 h (x, y, z p < 0.05). Duncan’s New Multiple-range test was used for the differences between the control group and two treatment groups at 0 h to 168 h. The capital letters represent stands the difference of the control group over time (A, B, C, D, E, F p < 0.05). Error bars indicate the standard errors of the mean.

To further verify the effects of the NAD^+^ and NADH treatments on postmortem glycolysis, the contents of muscle glycogen, free glucose, and lactic acid were determined. It can be seen from Table 1 that, with the extension of maturation time, the muscle glycogen content of the control group and the treatment groups decreased gradually (P < 0.05). During the 24–72 h maturation period, the muscle glycogen content of the NAD^+^ group was significantly lower than that of the control group (P < 0.05), while the muscle glycogen content of the NADH group was significantly higher than that of the control group. During the 120–168 h maturation period, the muscle glycogen content of the NAD^+^ group was lower than that of the control group, although the difference was not significant. At the same time, the muscle glycogen content in the NADH group was higher than that in the control group but the difference was not significant. The levels of free glucose in the control group and the treatment group remained essentially unchanged, and there were no significant differences among the three groups.

The lactate contents in the control and NAD^+^ groups first and then decreased (P < 0.05), reaching a maximum value after 72 h (P < 0.05). The lactate content in the NADH group also reached a maximum at 72 h and differed significantly from the control group. The lactic acid content after 24–168 h in the NAD^+^ group was significantly higher than that in the control group (P < 0.05). In the NAD^+^ group, myoglycolysis and lactate accumulation rate increased, while the lactate concentration in the NADH group increased by 108 ± 3.19 ng/ml at 72 h compared with that at 0 h, indicating that NADH inhibited the production of muscle lactic acid at the early postmortem stage.

### Changes in adenylate content in postmortem yak meat during aging

There was a continuous consumption of ATP during the maturation process while its production was further reduced. Postmortem muscle tissue lacks an energy supply and thus begins to disintegrate [22]. When the ATP is exhausted, ADP will release further energy and produce AMP. It can be seen from Table 2 that NAD^+^ increased the contents of skeletal muscle ATP while decreasing the AMP and IMP contents as the ripening time extended (P < 0.05), which may be due to NAD^+^ promotion of glycolysis. NAD^+^ activated glycolysis in the postmortem muscle, resulting in an increase of ATP production and an increase of ATP concentration within 12 h during aging. The IMP levels in postmortem yak meat were also significantly higher than the levels of AMP, which is consistent with previous results [23]. This finding again emphasizes that AMP is rapidly converted into IMP by deamination in postmortem skeletal muscle. There were no significant differences in the concentrations of adenylate among the three groups at 0 h. However, ATP production decreased and AMP and IMP increased by 12 h after NADH injection (P < 0.05). NADH inhibited glycolysis in the postmortem muscle, resulting in a decrease in ATP production at 12 h. The results showed that glycolysis in yak *longissimus thoracic* muscle decreased after NADH treatment, indicating that AMPK had a partial regulatory effect on glycolysis in the postmortem muscle [23].

**Table 2:** Nucleotide concentrations in postmortem yak *longissimus thoracic* muscle.

Time	0 h	12 h	24 h	72 h	120 h	168 h
**ATP contents (lmol/g muscle)**						
**NADH**	3.00 ± 0.083 ^ax^	1.69 ± 0.032 ^bx^	1.58 ± 0.032 ^cx^	0.11 ± 0.033 ^ex^	0.32 ± 0.087 ^dx^	0.45 ± 0.034 ^dx^
**Control**	3.01 ± 0.093 ^Ax^	1.95 ± 0.062 ^By^	1.84 ± 0.053 ^Cy^	0.22 ± 0.056 ^Ey^	0.51 ± 0.067 ^Dy^	0.61 ± 0.055 ^dy^
**NAD** ^**+**^	3.01 ± 0.041 ^ax^	2.26 ± 0.055 ^bz^	2.18 ± 0.051 ^cz^	0.57 ± 0.036 ^ez^	0.73 ± 0.025 ^dz^	0.81 ± 0.020 ^dz^
**ADP contents (lmol/g muscle)**						
**NADH**	3.91 ± 0.031 ^ax^	1.72 ± 0.046 ^bx^	0.035 ± 0.043 ^ex^	0.041 ± 0.072 ^dx^	0.57 ± 0.042 ^cx^	0.58 ± 0.021 ^cx^
**Control**	3.99 ± 0.026 ^Ax^	1.91 ± 0.073 ^By^	0.54 ± 0.070 ^Dy^	0.77 ± 0.112 ^Cy^	0.72 ± 0.087 ^Cy^	0.80 ± 0.025 ^Cy^
**NAD** ^**+**^	4.03 ± 0.042 ^az^	2.11 ± 0.045 ^bz^	0.89 ± 0.036 ^dz^	1.02 ± 0.078 ^cz^	1.05 ± 0.031 ^cz^	1.06 ± 0.055 ^cz^
**AMP contents (lmol/g muscle)**						
**NADH**	0.24 ± 0.011 ^ax^	0.09 ± 0.032 ^cx^	0.12 ± 0.028 ^bx^	0.065 ± 0.002 ^dx^	0.058 ± 0.005 ^ex^	0.031 ± 0.015 ^fx^
**Control**	0.25 ± 0.015 ^Ax^	0.14 ± 0.011 ^Cy^	0.18 ± 0.009 ^By^	0.11 ± 0.006 ^Dy^	0.087 ± 0.005 ^Ey^	0.077 ± 0.007 ^Fy^
**NAD** ^**+**^	0.25 ± 0.004 ^ax^	0.20 ± 0.010 ^cz^	0.25 ± 0.008 ^bz^	0.17 ± 0.003 ^dz^	0.104 ± 0.007 ^ez^	0.100 ± 0.003 ^fz^
**IMP contents (lmol/g muscle)**						
**NADH**	1.32 ± 0.021 ^fx^	1.96 ± 0.098 ^ex^	2.43 ± 0.121 ^dx^	5.42 ± 0.024 ^ax^	3.72 ± 0.021 ^cx^	4.15 ± 0.045 ^bx^
**Control**	1.34 ± 0.018 ^Fy^	2.28 ± 0.110 ^Ey^	2.67 ± 0.180 ^Dy^	5.74 ± 0.010 ^Ay^	4.05 ± 0.015 ^Cy^	4.38 ± 0.036 ^By^
**NAD** ^**+**^	1.34 ± 0.122 ^fz^	2.57 ± 0.105 ^ez^	2.84 ± 0.115 ^dz^	5.96 ± 0.012 ^az^	4.31 ± 0.002 ^cz^	4.56 ± 0.101 ^bz^

### Changes in AMPK activity in postmortem yak meat during aging

**Fig 1:**
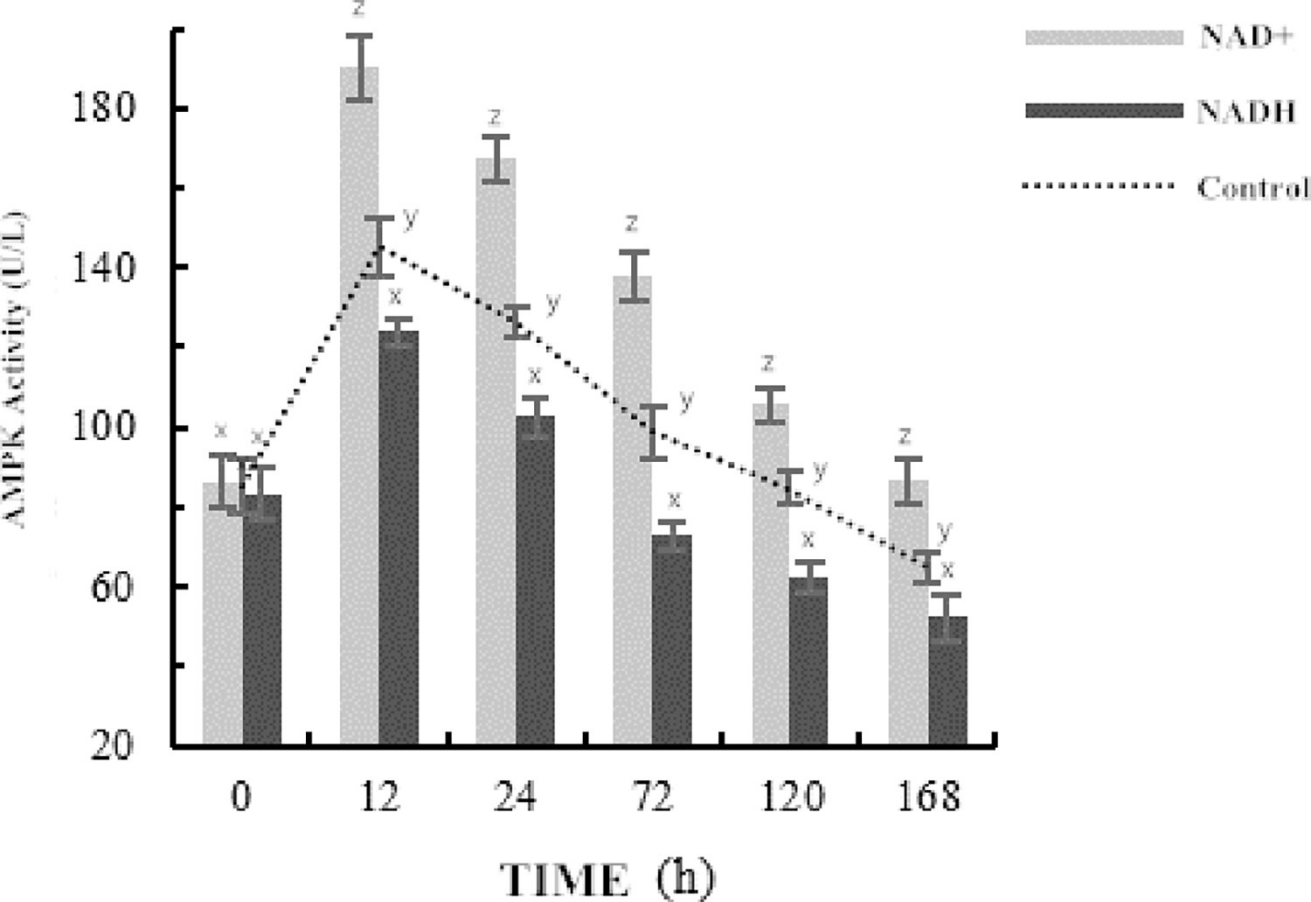
Changes of AMPK activity during post-mortem slaughter.

AMPK can regulate the balance of energy metabolism in cells and its activity can be used to judge the speed and degree of postmortem glycolysis [24]. It can be seen from Fig 1 that the AMPK activity in the control group and the NAD^+^ group increased first and then decreased as the ripening time extended, with the AMPK activity in the control group being significantly lower than that in the NAD^+^ group (P < 0.05). After 12 hours of maturation, the AMPK activity in the control group was 113.53 U/L, and that in the NAD^+^ group was 250.23 U/L. Compared with the control group, the activity of AMPK in the NAD^+^ group was increased by 49.23% (P < 0.05). After 168 h, the AMPK activity in the control group was 48.86 U/L, and that in the NAD^+^ group was 121.57 U/L. Compared with the control group, the activity of AMPK in the NAD^+^ group was increased by 59.81% (P < 0.05). However, NADH decreased significantly after 24 h, indicating that NADH reduced AMPK activity by regulating the NAD^+^/NADH balance.

### Investigation of the numbers and morphology of mitochondria and concentrations of PGC-1α

**Fig 2:**
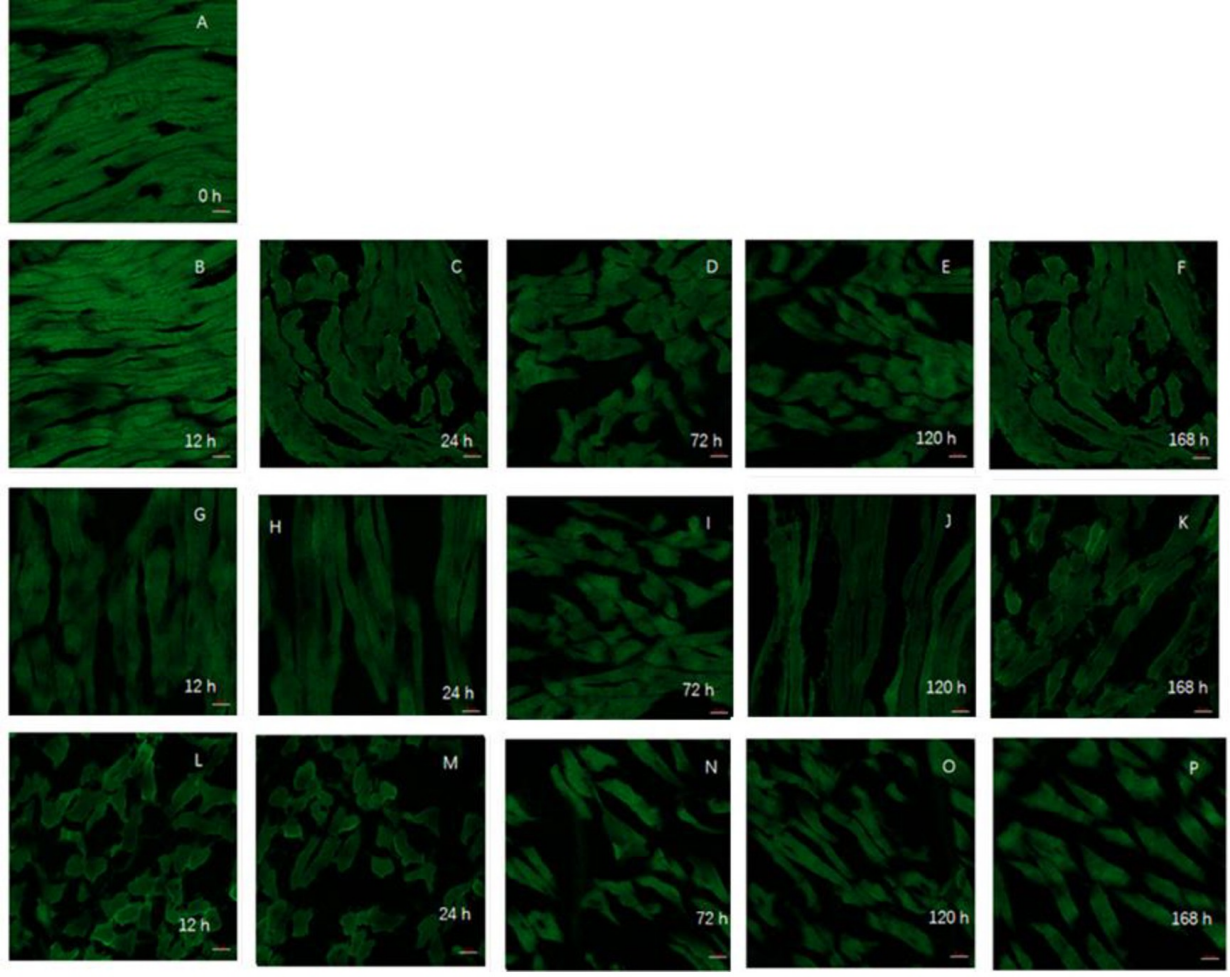
The morphological distribution of mitochondria in the muscle of the yak during postmortem maturation with a confocal laser microscope. The mitochondria are marked green by MitoTracker Green. A-F: at 0, 12, 24, 72, 120 and 168 h in the NAD^+^ treatment group; G-K: at 12, 24, 72, 120 and 168 h in the blank control group; L-P: at 12, 24, 72, 120 and 168 h in NADH treatment group.

Mitochondria are important organelles and are found in all eukaryotic cells, with the exception of mature red blood cells [17]. The main metabolic function of mitochondria is oxidative phosphorylation to provide energy for cells. Mitochondria are the center of cellular respiratory metabolism and are the main source of ATP [17]. Healthy mitochondria are closely related to the efficiency of cellular processes (Fig 2). The shape, number, size, and function of the mitochondria change in response to the environment. During the postmortem aging, mitochondria are one of the earliest organelles to reflect the trend of cell energy metabolism [3, 25]. In the early postmortem stages, the mitochondria in the control group were arranged regularly and the cristae were clear. There were no significant changes in the distribution and morphology of the mitochondria in myocytes between 0 h and 12 h. Between 12 h and 72 h, the mitochondria fused and enlarged, and the matrix became light, although the distribution remained regular. In the NAD^+^-treated group after 72 h and 120 h of maturation. However, the regular distribution of the mitochondria was completely lost after 72 h in the NADH-treated sample. The matrix became significantly lighter, the mitochondria expanded, and the cristae appeared to be completely dissolved with visible vacuoles, none of which was seen in either the NAD^+^-treated or control groups (see Fig 4).

**Fig 3:**
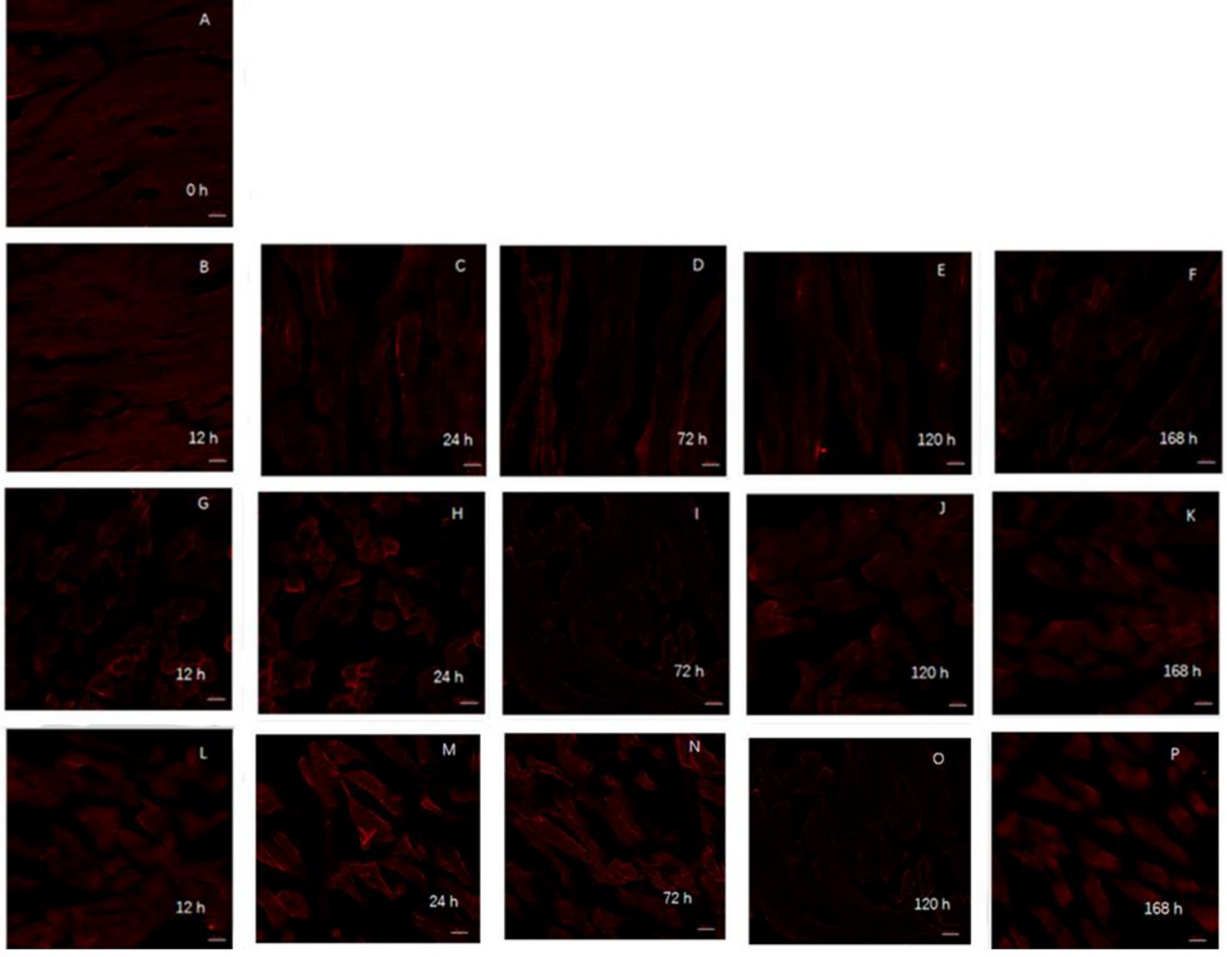
Observation of mitochondrial morphology distribution in yak muscle during postmortem maturation with laser confocal microscope. PGC1-α are marked by PGC1-α (1:100) in red. A-F: at 0, 12, 24, 72, 120 and 168 h in the NAD^+^ treatment group; G-K: at 12, 24, 72, 120 and 168 h in the blank control group; L-P: at 12, 24, 72, 120 and 168 h in NADH treatment group.

As a major regulator of mitochondrial biosynthesis, PGC-1α is an upstream gene that regulates ROS scavenging by proteins such as Mn SOD and UCP2 in the mitochondria [26]. Regulation of its expression may play an important role in reducing oxidative damage to the mitochondria and thus preventing mitochondrial dysfunction. It was observed that after prolonged maturation, the PGC-1α in the control group was arranged regularly with good morphology. The distribution and morphology of PGC-1α did not change significantly in the 0–12 h period (Fig 3). PGC-1α fusion occurred in the 12–72 h period, but the distribution was still regular. The distribution of PGC-1α was disordered in the NAD^+^ treated group after 120 h of postmortem maturation, and the changes of PGC-1α in the control group were similar to those in the NAD^+^ treated group at 72 h and 120 h after postmortem maturation. After NADH treatment, the regularity of the PGC-1α distribution was completely lost after 72 h of ripening, which was more serious than seen in either the NAD^+^-treatment group or the control group.

### Effects of NAD^+^ and NADH treatments on PGC-1α protein expression during postmortem yak meat maturation

**Fig 4:**
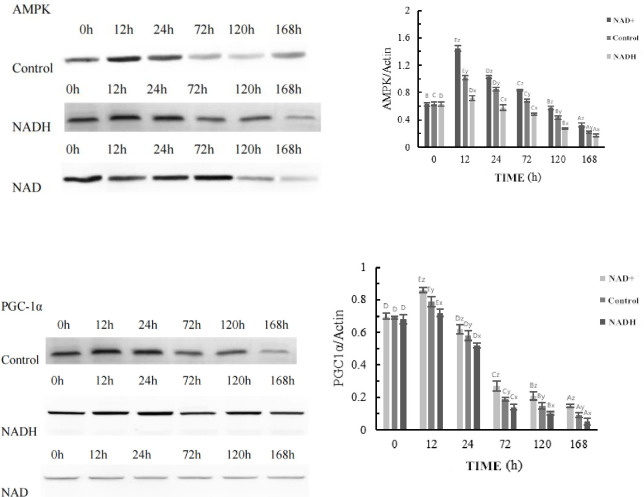
Representative western blot for effect of NAD^+^、NADH on AMPK and PGC1α expression.

The Western blotting and optical density analyses of PGC-1α and AMPK protein expression during the ripening process of postmortem yak meat treated with NAD^+^ and NADH are shown in Figs 3 and 4. Western blotting showed that the AMPK protein levels decreased gradually after 12 h in NAD^+^-treated myocytes, with the most significant decrease seen at 72 h (P < 0.01). The expression of NADH was increased at 12 h, after which it gradually decreased with prolongation of the ripening time. The difference was significant (P < 0.01, Fig 3). The expression of PGC-1α in the NAD^+^-treated group was slightly upregulated at 12 h compared with that at 0 h (P < 0.05). PGC-1α expression began to decrease at 24 h and continued to decrease with the progression of maturation (P < 0.01). The expression of NADH decreased slightly at 12 h compared with 0 h, but the difference was not significant (P > 0.05). The expression of NADH was decreased by 24 h and gradually decreased with the extension of maturation time (P < 0.01, Fig 4).

### Effects of NAD^+^ and NADH treatments on mitochondrial ROS levels during postmortem yak meat maturation

**Fig 5:**
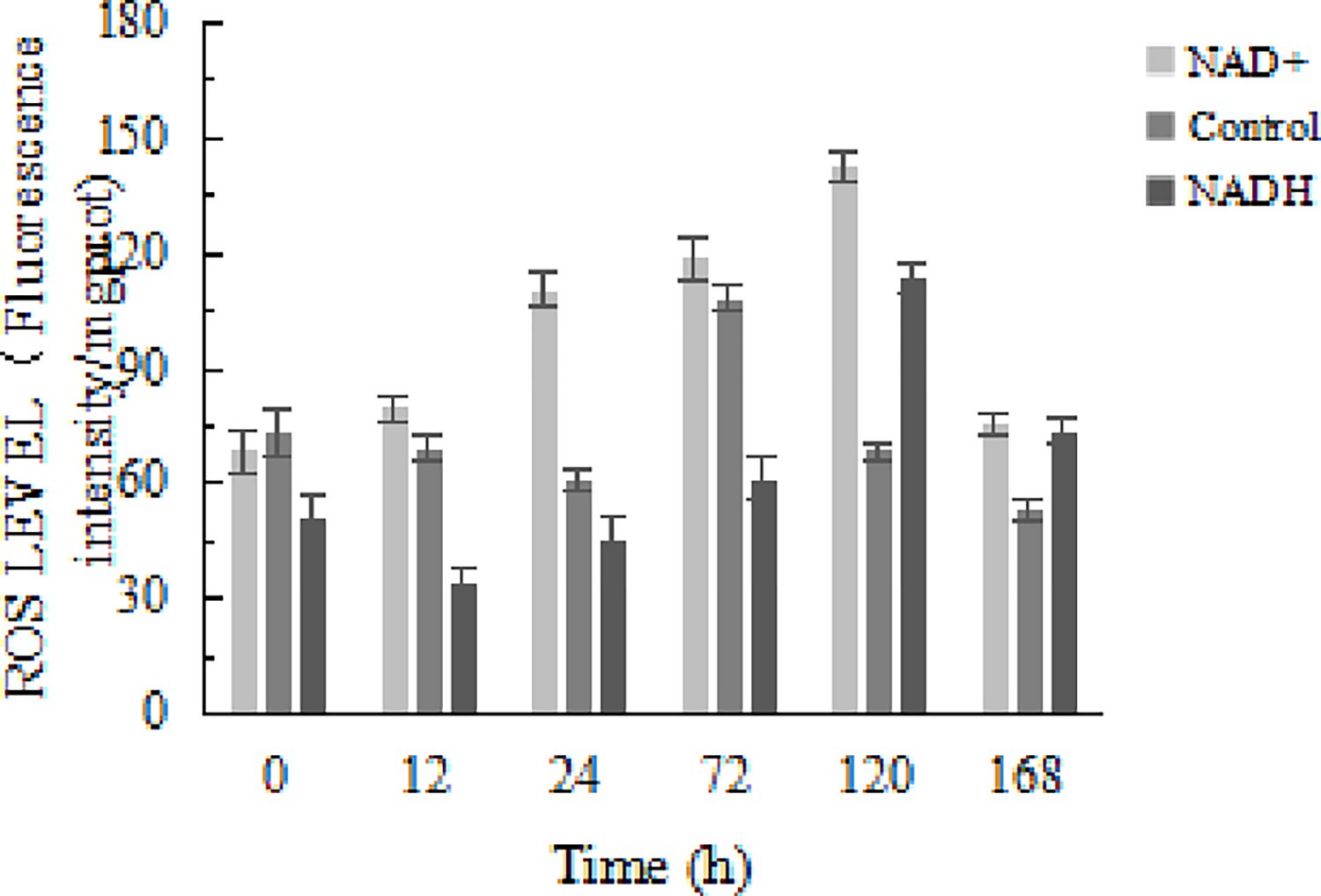
Effect of NAD^+^ and NADH on changes in mitochondrial ROS level.

ROS is mainly produced in the mitochondria. In addition to its direct damage to tissues, the ROS can cause further cellular damage through interaction with other factors and pathways [27]. As shown in Fig 5, the level of ROS in the NAD^+^-treatment group was significantly higher than that in the control and NADH-treatment groups (P < 0.01). The results show that NAD^+^ treatment can significantly increase the level of mitochondrial ROS, leading to an increase in the mitochondrial oxidative stress level. In the early period before 72 h, the ROS level in the control group was significantly higher than that in the NADH-treatment group (P < 0.01), indicating that the ROS scavenger NADH can inhibit oxidative stress in the mitochondria during postmortem aging.

### Effects of NAD^+^ and NADH treatments on muscle tenderness of postmortem yak meat during ripening

According to the analysis shown in Table 3, the MFI of the NAD^+^-treatment group was significantly higher than that of the NADH-treatment group (P < 0.01). The MFI of the NAD^+^-treatment group was significantly higher than that of the control group (P < 0.01) while the MFI of the control group was significantly higher than that of the NADH-treatment group at 12–168 h, with significant differences seen at the mid-mature stage (24–72 h). At 24 h after slaughter, the shear stress of the NAD^+^ treatment group was 9.73 Kgf, which was significantly higher than that of the control (13.98%) and the NADH-treatment groups (8.43%) (P < 0.01). During ripening, the shear stress of the NAD^+^-treatment group was significantly lower than that of the NADH treatment group (P < 0.01), indicating that NAD^+^ can further improve muscle tenderness by promoting glycolysis.

**Table 3:** Effect of NAD^+^ and NADH on changes in meat tenderness in yak LT muscle.

		0 h	12 h	24 h	72 h	120 h	168 h
**MFI**	**NAD** ^**+**^	40.60±3.87^A^	53.30±3.73^A^	71.28±5.44^A^	97.10±2.88^A^	106.82±6.64^A^	115.82±6.15^A^
	**Control**	39.46±0.96^A^	45.46±2.97^B^	59.32±4.86^B^	81.30±2.65^B^	92.82±4.35^B^	100.70±4.00^B^
	**NADH**	34.36±2.70^B^	39.68±3.64^B^	49.80±4.59^C^	70.16±3.29^C^	88.16±4.54^B^	100.80±3.06^B^
**Shear force (kgf)**	**NAD** ^**+**^	5.98±0.26^B^	6.85±0.42^B^	9.73±0.29^A^	8.64±0.56^C^	7.01±0.66^C^	4.55±0.61^B^
	**Control**	6.36±0.13^A^	7.79±0.34^A^	8.37±0.44^C^	9.80±0.42^B^	8.01±0.27^B^	5.39±0.62^A^
	**NADH**	6.43±0.24^A^	8.22±0.37^A^	8.91±0.45^B^	10.45±0.42^A^	9.11±0.53^A^	5.84±0.72^A^

## Conclusions

Changes in glycolysis, energy metabolism, AMPK activity, and AMPK and PGC-1α protein levels, as well as the numbers and morphology of the mitochondria, beef tenderness, and other related indices were measured during yak meat maturation. The results of this study demonstrate that SIRT1 expression and AMPK activity were dependent on NAD^+^ and NADH. This suggests that increased expression of SIRT1 will increase the activity of AMPK. Activation of AMPK leads to its phosphorylation and increased glycolytic activity, thus producing large amounts of lactic acid. This leads to reduced energy metabolism in postmortem animal muscles. Thus, AMPK activity was found to increase during adaptation to hypoxic conditions in yak meat, accelerating glycolysis and leading to greater regulation of energy production. These findings lay a foundation for the establishment of a theoretical system of energy metabolism in postmortem yak meat.

## Supporting information

S1 Raw images (PDF)
