# Supplementary figures and images for: Regulation of yak longissimus lumborum energy metabolism and tenderness by the AMPK/SIRT1 signaling pathways during postmortem storage
Source: PLoS One. 2022 Nov 28;17(11):e0277410. doi: 10.1371/journal.pone.0277410 (PMC9704559; doi:10.1371/journal.pone.0277410)

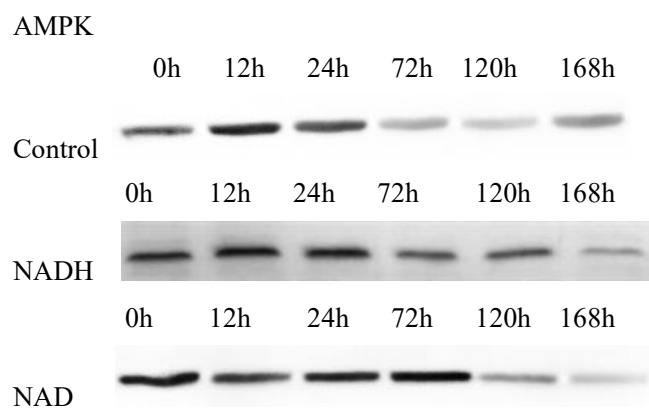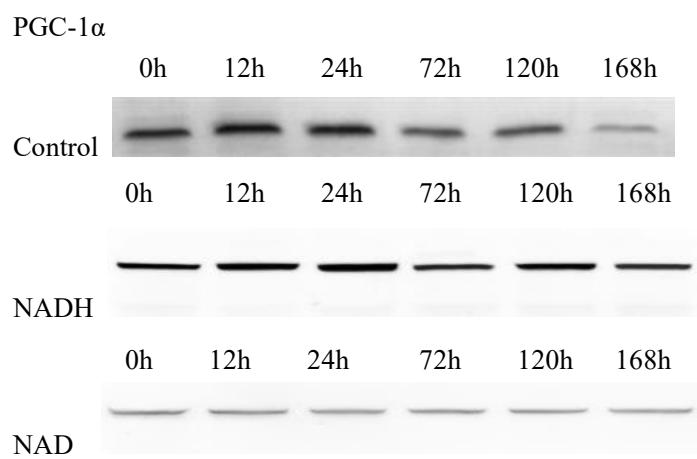

Supplement: S1 Raw images — (PDF) [file pone.0277410.s001.pdf]
